# Testcross Analysis of *Pl-1* Marker Expression and Seedling Vigor in Thai Maize Germplasm for Doubled Haploid Breeding Applications

**DOI:** 10.3390/plants14193011

**Published:** 2025-09-29

**Authors:** Arnat Thawarorit, Thomas Lübberstedt, Abil Dermail, Prakasit Duangpapeng, Vinitchan Ruanjaichon, Sompong Chankaew, Khundej Suriharn

**Affiliations:** 1 Department of Agronomy, Faculty of Agriculture, Khon Kaen University, Khon Kaen 40002, Thailand; ster_ast@hotmail.com (A.T.); abildermail@gmail.com (A.D.); somchan@kku.ac.th (S.C.); 2 Department of Agronomy, Iowa State University, Ames, IA 50011, USA; thomasl@iastate.edu; 3 Department of Horticulture, Faculty of Agriculture, Khon Kaen University, Khon Kaen 40002, Thailand; prakasit@kku.ac.th; 4 Plant Breeding Research Center for Sustainable Agriculture, Faculty of Agriculture, Khon Kaen University, Khon Kaen 40002, Thailand; 5 National Center for Genetic Engineering and Biotechnology (BIOTEC), 113 Thailand Science Park, Pahonyothin Road, Khlong Nueng, Khlong Luang, Pathum Thani 12120, Thailand; vinitchan.rua@biotec.or.th

**Keywords:** tropical maize, doubled haploid technology, haploid identification, root pigmentation, marker inheritance

## Abstract

Doubled haploid (DH) technology accelerates maize breeding by generating completely homozygous lines within two generations, but its efficiency depends on reliable haploid identification markers. The Purple plant 1 (*Pl-1*) root pigmentation marker has emerged as a promising alternative to R1-Navajo (*R1-nj*), which suffers from frequent suppression in tropical germplasm. This study evaluated *Pl-1* expression and seedling vigor in 298 diverse Thai maize genotypes across four market classes under controlled growth chamber conditions (24 ± 1 °C, 85–90% humidity, and standardized lighting), followed by testcross analysis with 89 representative genotypes crossed with BHI306 to distinguish between allelic absence and epistatic suppression mechanisms. Complete absence of *Pl-1* expression was observed in 99.3% of Thai genotypes, contrasting with consistent expression in the temperate-derived control (BHI306). Testcross F_1_ progeny from 89 Thai × BHI306 crosses exhibited intermediate expression levels (1.57–2.05) across all market classes, confirming allelic absence rather than suppressor-mediated inhibition. Substantial genetic diversity was detected in seedling vigor traits independent of *Pl-1* status, with root length varying 43-fold and fresh weight 20-fold, showing highly significant genotypic effects. The uniform lack of *Pl-1* expression across Thai germplasm eliminates background interference, highlighting its utility as a complementary marker when introgressed from inducer lines. These findings establish the genetic foundation for implementing optimized DH breeding strategies in tropical maize through marker-assisted backcrossing approaches.

## 1. Introduction

Maize (*Zea mays* L.) is a globally important crop for food, feed, and industry, with modern production relying on hybrid varieties that depend on high-quality parental inbred lines [1]. Thailand represents Southeast Asia’s largest maize producer, serving critical roles in feed industry and export markets while supporting rural livelihoods across diverse agroecological zones. Thai breeding programs must address unique tropical challenges, including heat stress, diverse market requirements, and rapid variety turnover demands, making accelerated breeding technologies particularly valuable. Conventional breeding requires 6–8 generations of self-pollination to achieve homozygosity, creating temporal constraints that limit genetic gain [2,3]. Doubled haploid (DH) technology addresses these limitations by producing completely homozygous lines in just two generations, dramatically accelerating breeding cycles and enabling more precise phenotypic evaluation [4,5].

The preferred in vivo DH method comprises maternal haploid induction, haploid identification, genome doubling, and self-pollination for DH seed production [4,6]. However, accurate identification of haploid kernels among predominantly diploid seeds remains a critical bottleneck [7]. The conventional *R1-nj* anthocyanin marker enables visual haploid identification through distinctive kernel pigmentation patterns [8,9], but its effectiveness in tropical maize breeding is severely limited by the high frequency (~50%) of the *C1-I* inhibitor gene in tropical germplasm [10,11].

Multiple approaches have been developed for haploid identification in maize [12]. Morphological methods rely on visual assessments of plant characteristics but require field evaluations and expert judgment. Cytological approaches provide definitive chromosome counts but demand specialized laboratory facilities. Flow cytometry and oil content analysis offer high-throughput screening but require expensive equipment and technical expertise. Anthocyanin-based visual markers remain the most practical approach for large-scale breeding programs due to their simplicity and cost-effectiveness, despite the expression limitations in tropical germplasm that this study aims to address.

The *R1-nj* gene encodes a transcriptional activator in the anthocyanin biosynthesis pathway that specifically activates aleurone pigmentation in maize kernels [8]. When functional, *R1-nj* induces purple pigmentation in the aleurone layer of developing kernels, allowing visual discrimination between haploid (unpigmented) and diploid (pigmented) seeds. However, the *C1-I* inhibitor gene, prevalent in tropical germplasm, produces a dominant inhibitor protein that blocks *R1-nj* transcriptional activity, resulting in colorless kernels regardless of *R1-nj* genotype [11]. In contrast, the *Pl-1* gene controls anthocyanin production in vegetative tissues, particularly root and stem tissues, through a different regulatory mechanism [13]. *Pl-1* encodes a MYB-type transcription factor that activates the anthocyanin biosynthetic pathway in root tissues during early seedling development. Unlike the kernel-based *R1-nj* system, *Pl-1* expression occurs in seedling roots 4–6 days after germination, enabling identification at the seedling stage rather than requiring seed maturation [13].

The dominant *Pl-1* gene controlling root pigmentation has emerged as a promising complementary system to overcome *R1-nj* limitations in tropical germplasm [13]. This marker enables visual discrimination between haploid and diploid seedlings based on anthocyanin coloration in roots, offering advantages such as early seedling-stage confirmation of haploid status, which is particularly valuable for resolving *R1-nj* misclassification issues caused by small embryos or *C1-I* inhibitor effects prevalent in tropical germplasm [10,14,15]. However, anthocyanin biosynthesis is regulated by complex networks subject to epistatic interactions (where inhibitor genes can suppress expression even when functional alleles are present) and environmental modulation (temperature, light, and stress effects) [16,17]. When marker expression is completely absent across diverse germplasm, controlled genetic crosses become essential to distinguish between the absence of functional alleles (producing intermediate F_1_ expression) versus the presence of epistatic suppressors that mask expression (maintaining suppression in F_1_ progeny) [15,18].

Complementary molecular marker approaches have been developed to address phenotypic marker limitations in tropical germplasm. Simple sequence repeat (SSR) markers linked to haploid-inducing genes enable early identification without relying on visual phenotypes [19]. Single-nucleotide polymorphism (SNP) arrays provide high-throughput screening capabilities for large-scale doubled haploid programs, particularly valuable when phenotypic markers are suppressed [20]. Quantitative trait loci (QTL) mapping has identified genomic regions associated with haploid induction rates in tropical backgrounds, enabling marker-assisted selection for improved inducer line development [21]. However, molecular approaches require laboratory infrastructure and technical expertise that may limit accessibility in resource-constrained breeding programs, making reliable phenotypic markers essential for widespread adoption of doubled haploid technology in developing regions [7].

The natural occurrence and inheritance patterns of the *Pl-1* marker in Thai maize populations remain unexplored, creating uncertainty about both its potential application and the genetic mechanisms governing its expression in tropical germplasm. Therefore, this research investigated *Pl-1* marker inheritance patterns through both observational screening and testcross analysis in diverse Thai maize germplasm, providing essential genetic insights for developing optimized haploid identification protocols tailored to tropical breeding programs.

## 2. Results

### 2.1. Expression of Pl-1 Marker in Thai Maize Germplasm

**Figure 1:**
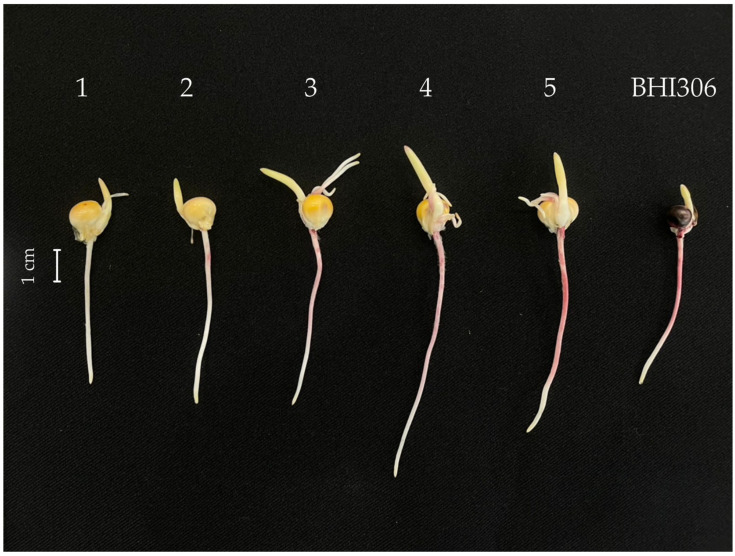
Visual scoring scale for *Pl-1* root pigmentation in maize seedlings 96 h after imbibition. Scale: 1 = intense purple/red coloration throughout the primary root; 2 = moderate purple/red coloration visible along the root length; 3 = light purple/red coloration detectable in root sections; 4 = very faint pigmentation barely visible to the naked eye; and 5 = complete absence of red/purple pigmentation. Scale bar = 1 cm.

Analysis of *Pl-1* marker expression revealed complete absence of anthocyanin pigmentation across all of the Thai maize germplasm in Experiment 1, while testcross analysis in Experiment 2 demonstrated clear inheritance patterns (Table 1 and Figure 1). In the germplasm screening, all 298 Thai genotypes exhibited uniform expression level 1 (complete absence of visible anthocyanin pigmentation) across all market categories, based on the 5-point visual scale, where 1 = complete absence of red/purple pigmentation; 2 = very faint pigmentation barely visible; 3 = light purple/red coloration detectable in root sections; 4 = moderate purple/red coloration visible along root length; and 5 = intense purple/red coloration throughout the primary root—field maize (*n* = 79), waxy maize (*n* = 98), small-ear waxy maize (*n* = 88), and sweet maize (*n* = 33). This contrasted with BHI306, which consistently exhibited expression level 5 (intense purple/red root coloration) across all replications.

In Experiment 1, all 298 Thai maize genotypes displayed expression level 1 (complete absence), while BHI306 controls showed level 5 (intense pigmentation). In Experiment 2, F_1_ testcross progeny exhibited intermediate expression levels (means 1.6–2.1 across market classes) between their Thai parents (1.0) and BHI306 parent (3.8–4.0). This demonstrated a clear distinction between the weak expression in the Thai germplasm and strong expression in the temperate-derived inducer line.

These results demonstrate that functional *Pl-1*-mediated anthocyanin biosynthesis is virtually absent in locally adapted Thai maize germplasm, regardless of market class or breeding background. The complete lack of intermediate expression levels suggests either the absence of the functional *Pl-1* allele or the presence of strong epistatic suppressors that completely inhibit anthocyanin production in root tissues under the experimental conditions employed.

Paradoxically, this uniform absence of *Pl-1* expression presents a significant operational advantage for haploid identification protocols within the Thai germplasm. The lack of background anthocyanin pigmentation eliminates potential false positives that could arise from endogenous color expression, thereby simplifying the visual discrimination between haploid (n) and diploid (2n) seedlings when *Pl-1*-expressing haploid inducer lines are employed. This genetic uniformity ensures that any detectable root pigmentation can be attributed exclusively to the inducer-derived *Pl-1* allele, enhancing the precision and reliability of haploid selection in doubled haploid breeding programs utilizing Thai germplasm.

### 2.2. Genetic Variation in Seedling Vigor Traits Across Maize Market Classes

Analysis of variance revealed highly significant genotypic effects (*p* ≤ 0.01) for both primary root length and fresh seedling weight measured 5 days after imbibition across all maize market categories evaluated (Table 2), demonstrating substantial genetic diversity in early seedling vigor traits independent of *Pl-1* marker expression status.

Waxy maize exhibited superior genetic parameters, with the highest mean squares (29.8 cm^2^ for root length; 1.60 g^2^ for fresh weight) and lowest coefficients of variation (5.3% and 9.4%, respectively), indicating strong genetic control and optimal experimental precision. Field maize showed comparable genetic variance with moderately low CVs (8.8% and 10.8%), while small-ear waxy maize and sweet maize displayed progressively higher experimental variation (CVs: 10.8–15.6%). The elevated variation in sweet maize likely reflects the different seed compositions and germination physiological characteristics of high-sugar genotypes.

The consistent detection of significant genotypic variation across all market classes indicates that seedling vigor traits are under independent genetic control and remain amenable to selection within the Thai germplasm. The substantial genetic variance observed (mean squares: 22.6–29.8 for root length) suggests considerable potential for genetic gain through conventional breeding approaches, providing alternative selection criteria for doubled haploid breeding programs where traditional markers may be ineffective.

### 2.3. Seedling Vigor Performance Within Market Classes Independent of Pl-1 Expression

Seedling vigor traits revealed extensive phenotypic diversity within Thai maize germplasm, with performance variation independent of *Pl-1* marker expression status (Table 3). Root length ranged from 0.3 cm (T89) to 12.9 cm (W84), representing a 43-fold difference, while fresh weight varied from 0.2 g (W1) to 4.1 g (W84), indicating substantial genetic diversity in early development capacity.

Top-performing genotypes included waxy maize W84 (12.9 ± 0.2 cm root length; 4.1 ± 0.1 g fresh weight) and field maize F66 (11.8 ± 0.2 cm; 3.4 ± 0.1 g) both classified as very high vigor. Several genotypes displayed dissociated trait expression, where root elongation and biomass accumulation did not correlate proportionally. For example, genotypes T89 and SC14 showed minimal root elongation (0.3–0.4 cm) while maintaining substantial biomass (1.0–1.7 g), suggesting independent genetic control of these developmental processes.

The BHI306 control demonstrated intermediate seedling vigor (5.3 ± 0.2 cm root length; 0.4 ± 0.2 g fresh weight), confirming that *Pl-1* expression does not inherently confer superior seedling performance. The observed phenotypic diversity within Thai germplasm demonstrates that seedling vigor traits represent independent selection targets, offering potential for genetic improvement through conventional breeding approaches even without functional haploid identification markers.

### 2.4. Market Class-Specific Performance Patterns in Seedling Vigor Traits

Statistical analysis revealed distinct performance hierarchies among Thai maize market classes for both seedling vigor traits (Table 4). Waxy maize exhibited superior performance with significantly higher mean root length (7.8 ± 1.3 cm) and fresh weight (1.6 ± 0.1 g) compared to all other categories. Field maize showed intermediate performance (5.8 ± 1.3 cm root length; 1.0 ± 0.1 g fresh weight), while small-ear waxy maize and sweet maize displayed progressively lower vigor metrics.

Coefficients of variation revealed differential genetic diversity patterns across market classes. Waxy maize displayed the most favorable combination of high mean performance and lowest variability (CV: 16.7% and 6.6%), indicating superior performance with good experimental precision. Sweet maize exhibited the highest variation (CV: 27.8% and 20.4%), likely reflecting either greater genetic diversity or increased sensitivity to experimental conditions due to altered seed physiology in high-sugar genotypes.

These patterns suggest market class-specific breeding strategies. Waxy maize offers immediate potential for elite line development, combining superior performance with consistent expression. Field maize, with an intermediate performance and large population size (*n* = 79), provides an opportunity for population improvement through recurrent selection. Small-ear waxy maize and sweet maize maintain sufficient genetic variation for targeted improvement within their market niches.

BHI306 performance (7.4 ± 0.2 cm root length; 0.9 ± 0.0 g fresh weight) approximated waxy maize root length but showed lower fresh weight, suggesting different resource-allocation patterns between temperate and tropical germplasm.

### 2.5. Independence of Seedling Vigor from Pl-1 Marker Expression

Comparative analysis between *Pl-1*-expressing and non-expressing genotypes demonstrated no statistically significant association between marker expression and seedling vigor traits (Table 5). The BHI306 control exhibited mean values of 7.4 ± 0.2 cm for root length and 0.9 ± 0.0 g for fresh weight, while Thai germplasm collectively averaged 5.9 ± 1.9 cm and 1.1 ± 0.7 g, respectively. Statistical testing revealed non-significant differences (t = 1.45, *p* > 0.05 for root length; t = 1.27, *p* > 0.05 for fresh weight).

This independence carries important implications for doubled haploid breeding strategies. The lack of association indicates that anthocyanin biosynthesis pathways and early growth vigor are under distinct genetic control mechanisms. Consequently, breeding programs can optimize these traits independently by selecting for robust seedling performance within Thai germplasm while separately introducing *Pl-1* marker functionality through backcrossing with temperate inducer lines.

### 2.6. Testcross Analysis Confirms Allelic Absence Rather than Epistatic Suppression

Testcross evaluation between 89 Thai genotypes and BHI306 revealed inheritance patterns consistent with allelic absence rather than epistatic suppression (Table 6). Complete individual genotype data for all 89 testcross entries are provided in Supplementary Materials (Table S1). F_1_ progeny showed *Pl-1* expression levels intermediate between Thai and BHI306 parents across all market classes. Field maize testcrosses exhibited mean *Pl-1* scores of 1.57 ± 0.48, waxy maize 2.05 ± 0.53, and sweet maize 1.64 ± 0.43, all significantly lower than the BHI306 parent (3.82 ± 0.30) but higher than typical Thai parent values. This inheritance pattern provides genetic evidence independent of environmental effects, as epistatic suppression would manifest as complete inhibition regardless of experimental conditions, while the observed intermediate expression demonstrates normal Mendelian inheritance of functional alleles.

The F_1_ generation displayed considerable variation in *Pl-1* expression within each market class (CV: 25.9–30.6%), with some individuals showing relatively strong expression (up to 4.2 in waxy maize), while others exhibited minimal pigmentation. This continuous variation pattern suggests that genetic background effects from Thai parents modulate *Pl-1* expression levels in F_1_ progeny beyond simple allelic dosage, indicating quantitative inheritance influenced by modifier loci or epistatic interactions with the BHI306-derived *Pl-1* allele.

Root length and fresh weight in F_1_ populations showed performance generally intermediate between parents. Mean root lengths ranged from 6.50 to 7.48 cm across market classes, falling between Thai parent ranges (4.9–7.8 cm from Table 4) and BHI306 performance (4.88 cm). Fresh weight values (1.30–1.61 g) were comparable to or slightly higher than those of Thai parents, indicating normal heterosis effects for vigor traits.

The intermediate *Pl-1* expression in F_1_ progeny, combined with the absence of complete suppression in any testcross combination, demonstrates that Thai germplasm lacks functional *Pl-1* alleles rather than carrying dominant suppressor genes. If epistatic suppressors were present, F_1_ individuals would show complete absence of pigmentation despite carrying the BHI306-derived dominant allele. This finding has important implications for breeding strategies, as it indicates that *Pl-1* marker functionality can be successfully introduced into Thai germplasm through conventional backcrossing approaches without interference from suppressor mechanisms.

## 3. Discussion

### 3.1. Genetic Mechanisms Underlying Pl-1 Expression in Thai Germplasm

Comprehensive evaluation of *Pl-1* root pigmentation marker expression across 300 diverse Thai maize genotypes revealed systematic absence of functional alleles throughout locally adapted germplasm. All Thai genotypes exhibited minimal *Pl-1* expression (scores 1.0) compared to the BHI306 temperate control (3.8–4.0) (Table 6), demonstrating a fundamental incompatibility between this marker system and tropical breeding populations. This limitation transcended market class boundaries, affecting field maize, waxy maize, small-ear waxy maize, and sweet maize equally, indicating that the absence of *Pl-1* functionality represents a characteristic feature of Thai-adapted germplasm rather than a market-specific phenomenon.

The testcross evaluation between Thai genotypes and BHI306 provided definitive resolution of the genetic mechanisms underlying *Pl-1* expression absence in tropical germplasm. Classical genetic theory predicts that dominant epistatic suppressor genes would completely inhibit marker expression even when functional alleles are present, while simple allelic absence would permit intermediate expression in heterozygous F_1_ progeny [18]. The intermediate *Pl-1* expression observed across all F_1_ populations (means 1.57–2.05) compared to parental values (Thai: 1.0; BHI306: 3.8–4.0) conclusively demonstrates allelic absence rather than suppressor-mediated inhibition. This inheritance pattern suggests absence of epistatic suppression, as dominant suppressor genes would have prevented expression in F_1_ individuals despite the presence of functional BHI306 alleles. Paradoxically, this uniform absence of *Pl-1* expression presents a significant operational advantage for haploid identification protocols within Thai germplasm. In breeding populations where some genotypes naturally express anthocyanin markers, distinguishing between inducer-derived pigmentation and background expression becomes challenging, leading to false-positive haploid identification that reduces selection accuracy. The complete lack of background anthocyanin pigmentation in Thai germplasm eliminates this interference, ensuring that any detectable root coloration in progeny from crosses with *Pl-1*-expressing inducer lines can be attributed exclusively to the inducer-derived *Pl-1* allele, effectively transforming what initially appears as a limitation into a strategic advantage for marker-based identification systems.

While minor suppressor effects cannot be completely excluded, the intermediate F_1_ expression patterns are most consistent with simple allelic absence combined with genetic background modulation rather than active suppression mechanisms.

While this study relied on phenotypic evaluation consistent with practical breeding applications, we acknowledge that molecular characterization would provide complementary evidence to support these findings. The controlled growth chamber conditions (24 ± 1 °C, 85–90% humidity, and standardized lighting) and rigorous experimental design minimized environmental confounding factors that could affect anthocyanin expression. The testcross inheritance patterns observed with F_1_ progeny showing intermediate expression levels (1.57–2.05) between parental extremes follow classical Mendelian genetics that distinguish allelic absence from suppressor mechanisms independent of environmental effects. Future comparative genomic analysis between Thai genotypes and *Pl-1* expressing lines would identify specific allelic variants affecting anthocyanin biosynthesis and enable the development of molecular markers for marker-assisted introgression programs.

The uniform absence of strong *Pl-1* expression contrasts with the partial suppression observed for *R1-nj* systems in tropical environments, where approximately 50% of genotypes retain some marker functionality [10,11]. The more severe limitation of *Pl-1* in Thai germplasm suggests systematic absence of functional alleles rather than suppressor mechanisms. The quantitative nature of *Pl-1* inheritance in F_1_ populations revealed additional complexity beyond simple Mendelian patterns. Continuous variation within testcross families (CV: 25.9–30.6%) suggests that expression levels are modulated by genetic background effects and modifier loci, consistent with findings in other anthocyanin biosynthesis studies [22].

This finding has profound implications for doubled haploid breeding strategies in tropical regions. While the absence of *Pl-1* functionality eliminates the possibility of developing locally adapted haploid induction systems using this marker, it also eliminates false-positive identification issues. Any detectable root pigmentation in progeny from crosses with *Pl-1*-expressing inducer lines can be attributed exclusively to inducer-derived genetic material, potentially improving selection accuracy compared to systems where background expression complicates identification [13]. The testcross approach successfully transformed preliminary observational findings into rigorous genetic analysis, addressing fundamental questions about inheritance mechanisms that cannot be resolved through screening alone.

### 3.2. Seedling Vigor Diversity and Independent Inheritance

Despite the uniform absence of *Pl-1* expression throughout Thai germplasm, extensive genetic variation was observed in seedling vigor traits across all evaluated genotypes. Root length exhibited remarkable diversity, spanning 43-fold variation from 0.3 to 12.9 cm, while fresh weight ranged 20-fold from 0.2 to 4.1 g, with highly significant genotypic effects detected across all traits (*p* ≤ 0.01). This substantial phenotypic diversity demonstrates that seedling vigor operates under independent genetic control from anthocyanin biosynthesis pathways, providing alternative selection targets for breeding programs where traditional haploid identification markers prove ineffective. The root length variation has direct implications for practical doubled haploid breeding programs—superior vigor genotypes (>10 cm root length) could serve as elite parents for developing robust doubled haploid lines that establish quickly under field conditions, while genotypes with poor early vigor (<2 cm root length) should be avoided as recurrent parents or targeted for improvement before doubled haploid conversion. The independent inheritance of vigor traits from *Pl-1* expression enables breeding programs to simultaneously select for both robust seedling establishment and marker functionality through parallel selection strategies, significantly improving doubled haploid program efficiency by eliminating weak genotypes early in the breeding cycle.

Market class comparisons revealed distinct performance hierarchies, with waxy maize exhibiting superior mean performance (7.8 ± 1.3 cm root length; 1.6 ± 0.1 g fresh weight) coupled with the lowest variability (CV: 16.7% and 6.6%), indicating superior performance with good experimental precision. Field maize showed intermediate performance, while sweet maize displayed the highest variation (CV: 27.8% and 20.4%), likely reflecting either greater genetic diversity or increased sensitivity to experimental conditions due to altered seed physiology in high-sugar genotypes. The magnitude of genetic variation observed in Thai germplasm equals or exceeds that reported in temperate breeding populations, indicating that tropical adaptation has not compromised genetic potential for early seedling performance [3,23].

Comparative analysis between *Pl-1*-expressing and non-expressing genotypes demonstrated no statistically significant association between marker expression and seedling vigor traits. The BHI306 control exhibited mean values of 7.4 ± 0.2 cm for root length and 0.9 ± 0.0 g for fresh weight, while Thai germplasm collectively averaged 5.9 ± 1.9 cm and 1.1 ± 0.7 g, respectively. Statistical testing revealed non-significant differences (t = 1.45, *p* > 0.05 for root length; t = 1.27, *p* > 0.05 for fresh weight). This independence was confirmed by testcross analysis, where F_1_ populations showed normal quantitative inheritance patterns for seedling traits regardless of anthocyanin expression levels.

Several genotypes displayed dissociated trait expression, where root elongation and biomass accumulation patterns did not correlate proportionally, suggesting independent genetic control of different developmental processes during early seedling establishment. This dissociation offers opportunities for targeted selection approaches and establishes Thai germplasm as a valuable genetic resource for enhancing seedling vigor in doubled haploid breeding programs. The substantial genetic variance observed across market classes suggests considerable potential for genetic gain through conventional breeding approaches, enabling breeding programs to optimize early plant establishment characteristics while simultaneously addressing haploid identification challenges through parallel selection strategies [24,25].

### 3.3. Strategic Implementation and Breeding Applications

The absence of functional *Pl-1* alleles in Thai germplasm presents both challenges and opportunities for implementing doubled haploid technology in tropical breeding programs. Three strategic pathways emerge for Thai breeding programs based on the testcross findings. First, direct adoption of temperate-derived inducer lines like BHI306 provides immediate access to *Pl-1*-based haploid identification, though adaptation to tropical conditions would need to be evaluated under field conditions. The intermediate F_1_ expression observed across all market classes demonstrates that this approach is genetically feasible, with *Pl-1* functionality expressing consistently despite diverse Thai genetic backgrounds.

Second, systematic introgression of *Pl-1* alleles into locally adapted backgrounds through marker-assisted backcrossing offers a more sustainable long-term solution. The quantitative inheritance patterns observed in F_1_ populations suggest that multiple backcross generations may be required to achieve optimal expression levels, but the absence of suppressor genes eliminates major genetic obstacles to this approach. This strategy enables the development of tropically adapted inducer lines while maintaining *Pl-1* marker functionality [3,23]. Cost–benefit analysis suggests that marker-assisted introgression represents the most viable approach for large-scale implementation. While requiring initial investment in molecular breeding infrastructure, this strategy enables the development of locally adapted inducer lines that combine tropical performance with reliable haploid identification, supporting sustainable doubled haploid breeding programs tailored to Thai growing conditions and market requirements.

Third, the uniform absence of background *Pl-1* expression provides unexpected operational advantages for haploid identification accuracy. Any detectable root pigmentation in progeny from crosses with *Pl-1*-expressing inducers can be attributed exclusively to inducer-derived genetic material, effectively eliminating false-positive identifications that complicate marker systems in populations with variable background expression [13]. The independent inheritance of seedling vigor traits offers additional flexibility for breeding program optimization. Programs can simultaneously select for robust early seedling performance within Thai germplasm while introducing *Pl-1* marker functionality, enabling the development of elite doubled haploid lines with both superior agronomic performance and reliable haploid identification capabilities [4,14].

Priority research should focus on comparative genomic analysis targeting several key regions: (1) the *Pl1* locus itself on chromosome 6 [13] to identify sequence variants, deletions, or insertions that prevent functional protein production; (2) regulatory elements upstream and downstream of *Pl1*, including promoter regions and enhancer sequences that control tissue-specific expression; (3) anthocyanin biosynthesis pathway genes, including *C1*, *C2*, *A1*, *A2*, *Bz1*, and *Bz2*, that interact epistatically with *Pl1* [16,17]; (4) transcription factor genes (*R*, *B*, and *Lc*) that regulate the anthocyanin pathway and could modify *Pl1* expression [16]; and (5) potential suppressor loci identified through QTL mapping in segregating populations [26]. Whole-genome sequencing comparisons between high-expressing temperate lines (BHI306) and Thai genotypes would identify structural variants, while RNA-seq analysis in root tissues could reveal expression differences in pathway components. Additionally, GWAS analysis using diverse tropical germplasm crossed with *Pl1*-expressing lines could identify modifier loci affecting expression levels observed in our F_1_ populations (CV: 25.9–30.6%) [27]. These genomic approaches would enable the development of diagnostic markers for marker-assisted introgression and help predict which Thai backgrounds are most suitable for successful *Pl1* integration.

The development of molecular markers linked to *Pl-1* functionality represents a critical need for marker-assisted introgression programs, enabling early-generation selection and accelerating backcrossing efficiency in developing locally adapted inducer lines [15]. Large-scale validation studies are essential before implementing doubled haploid technology in Thai breeding programs, including evaluation across multiple environments and assessment of haploid induction rates using locally adapted versus temperate-derived inducer lines. Additionally, field validation studies under diverse tropical environments represent an important next step for confirming the practical utility of these findings in operational doubled haploid breeding programs. Such studies should evaluate Pl-1 expression consistency across multiple locations, seasons, and stress conditions to establish robust protocols for tropical breeding applications.

## 4. Materials and Methods

### 4.1. Plant Materials

A diverse panel of 298 tropical maize genotypes was assembled to represent Thailand’s four major market segments: field maize (*n* = 79), waxy maize (*n* = 98), small-ear waxy maize (*n* = 88), and sweet maize (*n* = 33). Both waxy maize types carry the same waxy gene (*wx*) but differ in ear- and seed-size characteristics—conventional waxy maize (large ears, 15–20 cm) versus small-ear waxy maize (compact ears, 8–12 cm) bred for fresh market consumption. All genotypes were locally adapted through multiple generations of selection under tropical conditions and were obtained from the Plant Breeding Research Center for Sustainable Agriculture, Khon Kaen University. BHI306, a temperate-derived haploid inducer line known to exhibit stable *Pl-1* marker expression, served as the positive control and male parent for testcross analysis.

For testcross evaluation, 89 representative Thai genotypes were selected from the original collection, comprising field maize (*n* = 30), waxy maize (*n* = 29), and sweet maize (*n* = 30), and crossed with two independent BHI306 sources as male parents. This subset was chosen to provide balanced representation across major market classes while maintaining experimental feasibility. The germplasm collection was specifically curated to capture maximum genetic diversity within Thai maize breeding programs and enable a comprehensive evaluation of *Pl-1* marker inheritance patterns through both direct screening and controlled genetic analysis.

### 4.2. Testcross Development

Testcrosses were developed during the 2023 growing season at the Field Crop Research Station, Khon Kaen University. A total of 89 representative Thai genotypes were selected as female parents and crossed with BHI306, which served as the male parent (pollen donor). Crosses were conducted using standard hand-pollination techniques: female plants were covered with pollination bags 2–3 days before silk emergence to prevent contamination, silks were hand-pollinated with fresh pollen collected from BHI306 tassels in the morning (8:00–11:30 a.m.), and ears were re-bagged immediately after pollination. Each cross was replicated using multiple ears per genotype to ensure adequate F_1_ seed production.

Field plots consisted of single 5 m rows with 25 cm plant spacing and 75 cm row spacing, with 20 plants per row. Two rows were dedicated to each genotype. Pollination success was monitored, and F_1_ seeds were harvested at physiological maturity, dried to 12–14% moisture content, and stored under controlled conditions until laboratory evaluation. The resulting F_1_ testcross seeds were subsequently evaluated in controlled laboratory conditions, as described in Section 4.3, with 30 seeds per testcross combination per replication arranged in germination boxes for *Pl-1* expression and seedling vigor assessment.

### 4.3. Laboratory Experiment

Two complementary experiments were conducted in controlled laboratory conditions during the 2024 dry season at the Seed Technology Laboratory, Field Crop Research Station, Khon Kaen University, Thailand (16°28′27.7″ N, 102°48′36.5″ E, 190 m above sea level). Both experiments employed a randomized complete block design (RCBD) with four replications, where each replication represented a separate germination chamber to control for potential environmental variation. Controlled growth chamber conditions were specifically chosen to minimize environmental factors that could affect anthocyanin expression and ensure accurate genetic interpretation of *Pl-1* inheritance patterns, following established protocols for haploid identification research.

Experiment 1: Germplasm screening evaluated all 300 Thai maize genotypes plus BHI306 as positive control to assess baseline *Pl-1* expression patterns across the diverse collection and establish the scope of marker absence in tropical germplasm.

Experiment 2: Testcross analysis evaluated F_1_ seeds from crosses between 89 representative Thai genotypes (field maize (*n* = 30), waxy maize (*n* = 29), and sweet maize (*n* = 30)) and two independent BHI306 sources, totaling 178 testcross combinations plus parental controls. This experiment was specifically designed to distinguish between two competing hypotheses: (1) absence of functional *Pl-1* alleles versus (2) presence of epistatic suppressors masking expression, thereby providing definitive genetic evidence for the mechanisms underlying *Pl-1* expression patterns in tropical germplasm.

### 4.4. Seed Preparation and Germination Setup

For Experiment 1 (germplasm screening), 30 uniform seeds per genotype per replication were arranged in three rows of 10 seeds each on sterile germination paper (Anchor Paper Co., Saint Paul, MN, USA, 76 lb basis weight, 5 × 10 cm^2^). For Experiment 2 (testcross analysis), 30 F_1_ seeds per testcross combination per replication were arranged in three rows of 10 seeds each on the same germination paper and under the same specifications. All germination papers were moistened with 15 mL of sterile distilled water and placed in sealed germination boxes to maintain humidity. Seeds were surface-sterilized with 1% sodium hypochlorite solution for 2 min and rinsed three times with sterile distilled water before placement to minimize contamination risk.

### 4.5. Growth Chamber Conditions

Germination boxes were incubated in programmable growth chambers (Model ECONOMIC DELUX MODEL ECD01E; Tilburg, The Netherlands) maintained at 24 ± 1 °C with complete darkness for 96 h. Temperature control was achieved using a Jumo d’TRON 304 controller (JUMO GmbH & Co. KG, Fulda, Germany) with ±0.3 °C precision. Relative humidity was maintained at 85–90% through saturated paper towels placed in the chamber. Temperature and humidity were monitored continuously using data loggers, and chambers were rotated daily to minimize positional effects.

### 4.6. Red Root Assessment

Root pigmentation evaluation was conducted under standardized lighting conditions (fluorescent white light, 1000 lux) at exactly 96 h post-imbibition. Each seedling was individually examined using the 5-point visual scale (Figure 1): score 1 = intense purple/red coloration throughout the primary root; score 2 = moderate purple/red coloration visible along the root length; score 3 = light purple/red coloration detectable in root sections; score 4 = very faint pigmentation barely visible to the naked eye; and score 5 = complete absence of red/purple pigmentation. Assessment was performed by a single trained evaluator to ensure consistency.

### 4.7. Seedling Vigor Assessment

Seedling vigor was quantified through two traits: primary root length (cm) and fresh seedling weight (g). All measurements were recorded at the individual seedling level. Data collection protocols ensured minimal handling stress and rapid processing to maintain measurement accuracy.

### 4.8. Statistical Analysis

For both experiments, data were analyzed according to the randomized complete block design model *Yij* = *μ* + *Gi* + *Bj* + *εij*, where genotype effects (*Gi*) were fixed and block effects (*Bj*) were random. Analysis of variance was performed separately for each genotype category and combined across categories. Mean comparisons employed Fisher’s LSD test (*p* ≤ 0.05). Statistical procedures followed [28] methodology for agricultural research. All statistical analyses were conducted using Statistix 10 software (Analytical Software, Tallahassee, FL, USA).

## 5. Conclusions

This study revealed complete absence of *Pl-1* expression across 298 Thai maize genotypes, with testcross analysis confirming allelic absence rather than epistatic suppression. F_1_ progeny showed intermediate expression levels (1.57–2.05) across all market classes, ruling out suppressor mechanisms and validating marker introgression potential. This uniform absence eliminates false-positive identification when *Pl-1*-expressing inducer lines are employed while enabling development of locally adapted induction systems through backcrossing. Substantial genetic diversity in seedling vigor traits revealed 43-fold variation in root length and 20-fold variation in fresh weight, independent of *Pl-1* status. While haploid performance data would strengthen vigor-based selection conclusions, the extensive phenotypic diversity suggests potential for genotype-specific identification protocols. The testcross methodology successfully distinguished between competing genetic hypotheses, transforming preliminary observations into rigorous genetic analysis. While molecular validation would strengthen these phenotypic findings, the controlled experimental conditions and classical inheritance patterns observed in testcross analysis provide robust genetic evidence for the mechanisms underlying *Pl-1* expression in tropical germplasm. These findings establish *Pl-1* marker utility in tropical germplasm as a strategic opportunity rather than limitation, enabling parallel selection for identification accuracy and genetic improvement in doubled haploid breeding programs tailored to tropical conditions.

## Figures and Tables

**Table 1:** Frequency and percentage distribution of *Pl-1* marker expression levels across different maize genotype categories.

Genotype Category	No. of Lines	Expression Level				
		Level 5 (Very Dark)	Level 4 (Dark)	Level 3 (Medium)	Level 2 (Light)	Level 1 (No Expression)
Field maize	79	0 (0.0%)	0 (0.0%)	0 (0.0%)	0 (0.0%)	79 (100.0%)
Waxy maize	98	0 (0.0%)	0 (0.0%)	0 (0.0%)	0 (0.0%)	98 (100.0%)
Small-ear waxy maize	88	0 (0.0%)	0 (0.0%)	0 (0.0%)	0 (0.0%)	88 (100.0%)
Sweet maize	33	0 (0.0%)	0 (0.0%)	0 (0.0%)	0 (0.0%)	33 (100.0%)
BHI306 (control)	1	1 (100.0%)	0 (0.0%)	0 (0.0%)	0 (0.0%)	0 (0.0%)
Total	299	1 (0.3%)	0 (0.0%)	0 (0.0%)	0 (0.0%)	298 (99.7%)

Expression levels were assessed using a 1–5 scale, where 5 = intense purple/red root coloration and 1 = absence of pigmentation. BHI306 served as a positive control line known to express the *Pl-1* marker.

**Table 2:** Analysis of variance for seedling root length and fresh weight 5 days after imbibition across different maize genotype categories.

Genotype Category	Source of Variation	Df	Mean Square	
			Root Length (cm^2^)	Fresh Weight (g^2^)
Field maize	Genotype	79	27.9 **	1.44 **
(*n* = 79)	Error	237	0.3	0.01
	CV (%)		8.8	9.7
Waxy maize	Genotype	98	29.8 **	1.60 **
(*n* = 98)	Error	294	0.2	0.01
	CV (%)		5.3	6.2
Small-ear waxy maize	Genotype	88	22.6 **	0.59 **
(*n* = 88)	Error	264	0.3	0.01
	CV (%)	-	10.8	11.1
Sweet maize	Genotype	33	23.0 **	0.51 **
(*n* = 33)	Error	97	0.2	0.01
	CV (%)	-	13.7	14.3

** Statistically significant at *p* ≤ 0.01; CV = coefficient of variation.

**Table 3:** Seedling characteristics of representative maize genotypes showing *Pl-1* marker expression variation.

Genotype	Category	*Pl-1* Expression Level	Root Length (cm)	Fresh Weight (g)	Vigor Class ^1^
F66	Field maize	1 (No expression)	11.8 ± 0.2	2.1 ± 0.1	Very high
F42	Field maize	1 (No expression)	1.1 ± 0.1	0.1 ± 0.2	Very low
W84	Waxy maize	1 (No expression)	12.9 ± 0.2	2.2 ± 0.1	Very high
W1	Waxy maize	1 (No expression)	0.6 ± 0.1	0.2 ± 0.2	Very low
T15	Small-ear waxy maize	1 (No expression)	9.7 ± 0.2	2.0 ± 0.1	High
T89	Small-ear waxy maize	1 (No expression)	0.3 ± 0.1	0.1 ± 0.1	Very low
SC25	Sweet maize	1 (No expression)	9.5 ± 0.2	0.9 ± 0.0	High
SC14	Sweet maize	1 (No expression)	0.4 ± 0.1	0.3 ± 0.0	Low
BHI306-1	Positive control	5 (Very dark)	5.3 ± 0.2	0.4 ± 0.2	Moderate

Values represent means ± standard error from three replications. ^1^ Vigor classification: very low (<2 cm), low (2–4 cm), moderate (4–7 cm), high (7–10 cm), and very high (>10 cm).

**Table 4:** Distribution of seedling root length and fresh weight across different maize genotype categories.

Genotype Category	No. of Lines	Root Length (cm)			Fresh Weight (g)		
		Range	Mean ± SE	CV (%)	Range	Mean ± SE	CV (%)
Field maize	79	3.5–8.9	5.8 ± 1.3 ^b^	22.4	0.9–1.0	1.0 ± 0.1 ^b^	10.4
Waxy maize	98	3.8–9.5	7.8 ± 1.3 ^a^	16.7	1.5–1.7	1.6 ± 0.1 ^a^	6.6
Small-ear waxy maize	88	3.2–7.8	4.9 ± 1.1 ^c^	22.4	0.7–0.8	0.7 ± 0.1 ^c^	11.9
Sweet maize	33	2.0–5.5	3.6 ± 1.0 ^d^	27.8	0.4–0.5	0.5 ± 0.1 ^c^	20.4
BHI306 (control)	1	7.2–7.5	7.4 ± 0.2 ^a^	2.7	0.9–0.9	0.9 ± 0.0 ^ab^	3.7
Overall	299	2.0–9.5	5.9 ± 1.9	32.2	0.0–3.0	1.1 ± 0.7	64.9

Values represent means ± standard error; CV = coefficient of variation; different letters indicate significant differences (LSD test, *p* ≤ 0.05).

**Table 5:** Comparison of seedling traits between maize genotypes with and without *Pl-1* marker expression.

Trait	*Pl-1*-Expressing Genotypes		Non-Expressing Genotypes		t-Value
	N	Mean ± SE	n	Mean ± SE	
Root length (cm)	1 ^1^	7.2 ± 0.1	299	5.9 ± 0.1	1.45 ns
Fresh weight (g)	1 ^1^	2.7 ± 0.1	299	2.3 ± 0.0	1.27 ns

Values represent means ± standard error; ^1^ single genotype (BHI306); ns = not significant (*p* > 0.05).

**Table 6:** Testcross performance of F_1_ progeny from crosses between Thai maize genotypes and BHI306.

Genotype Category		Field Maize (*n* = 30)	Waxy Maize (*n* = 29)	Sweet Maize (*n* = 30)	BHI306 (*n* = 2)
*Pl-1* score	Range	0.3–2.7	1.0–4.2	0.47–2.87	3.36–4.27
	Mean ± SD	1.57 ± 0.48	2.05 ± 0.53	1.64 ± 0.43	3.82 ± 0.30
	CV (%)	30.6	25.9	26.2	7.9
Root length (cm)	Range	1.1–11.5	2.0–10.81	2.15–11.55	4.40–5.33
	Mean ± SD	6.50 ± 1.94	7.48 ± 1.74	6.59 ± 2.21	4.88 ± 0.29
	CV (%)	29.8	23.3	33.5	5.9
Fresh weight (g)	Range	0.2–2.5	0.17–3.22	0.35–2.51	0.40–0.53
	Mean ± SD	1.30 ± 0.57	1.61 ± 0.54	1.31 ± 0.56	0.48 ± 0.04
	CV (%)	43.8	33.5	42.7	8.3

Values represent means ± standard deviation; CV = coefficient of variation; *Pl-1* expression assessed using 5-point visual scale where 5 = intense purple/red root coloration and 1 = complete absence of pigmentation [13].

## Data Availability

The original contributions presented in this study are included in the article/Supplementary Materials. Further inquiries can be directed to the corresponding author.

## Supplementary Materials

The following supporting information can be downloaded at https://www.mdpi.com/article/10.3390/plants14193011/s1, Table S1: Individual genotype performance for *Pl-1* expression and seedling vigor traits.

## Abbreviations

The following abbreviations are used in this manuscript:

DH	Doubled haploid
F_1_	First filial generation (offspring from crossing two parental lines)
*C1-I*	Inhibitor gene that suppresses *R1-nj* anthocyanin marker expression
*Pl-1*	Purple plant gene controlling root pigmentation in maize

## References

[1] Troyer A.F., Bennetzen J.L., Hake S. (2009). Development of Hybrid Corn and the Seed Corn Industry. Handbook of Maize: Genetics and Genomics.

[2] Sleper D.A., Poehlman J.M. (2006). Breeding Field Crops.

[3] Bernardo R. (2014). Essentials of Plant Breeding.

[4] Chaikam V., Molenaar W., Melchinger A.E., Boddupalli P.M. (2019). Doubled haploid technology for line development in maize: Technical advances and prospects. Theor. Appl. Genet..

[5] Dermail A., Chankaew S., Lertrat K., Lübberstedt T., Suriharn K. (2021). Selection gain of maize haploid inducers for the tropical savanna environments. Plants.

[6] Geiger H.H., Gordillo G.A. (2009). Doubled haploids in hybrid maize breeding. Maydica.

[7] Prasanna B.M., Chaikam V., Mahuku G. (2012). Doubled Haploid Technology in Maize Breeding: Theory and Practice.

[8] Nanda D.K., Chase S.S. (1966). An embryo marker for detecting monoploids of maize (*Zea mays* L.). Crop Sci..

[9] Chaikam V., Prasanna B.M., Prasanna B.M., Chaikam V., Mahuku G. (2012). Maternal haploid induction in maize using *R1-nj* marker. Doubled Haploid Technology in Maize Breeding: Theory and Practice.

[10] Chaikam V., Lopez L.A., Martinez L., Burgueño J., Boddupalli P.M. (2017). Identification of in vivo-induced maternal haploids in maize using seedling traits. Euphytica.

[11] Lopez L.A., Ochieng J., Pacheco M., Martinez L., Omar H.A., Gowda M., Prasanna B.M., Dhugga K.S., Chaikam V. (2023). Effectiveness of *R1-nj* anthocyanin marker in the identification of in vivo induced maize haploid embryos. Plants.

[12] Dermail A., Mitchell M., Foster T., Fakude M., Chen Y.-R., Suriharn K., Frei U.K., Lübberstedt T. (2024). Haploid identification in maize. Front. Plant Sci..

[13] Chaikam V., Martinez L., Melchinger A.E., Schipprack W., Boddupalli P.M. (2016). Development and validation of red root marker-based haploid inducers in maize. Crop Sci..

[14] Trentin H.U., Frei U.K., Lübberstedt T. (2020). Breeding maize maternal haploid inducers. Plants.

[15] Chaikam V., Nair S.K., Babu R., Martinez L., Tejomurtula J., Boddupalli P.M. (2015). Analysis of effectiveness of *R1-nj* anthocyanin marker for in vivo haploid identification in maize and molecular markers for predicting the inhibition of *R1-nj* expression. Theor. Appl. Genet..

[16] Dooner H.K., Robbins T.P., Jorgensen R.A. (1991). Genetic and developmental control of anthocyanin biosynthesis. Annu. Rev. Genet..

[17] Goff S.A., Cone K.C., Chandler V.L. (1992). Functional analysis of the transcriptional activator encoded by the maize B gene: Evidence for a direct functional interaction between two classes of regulatory proteins. Genes Dev..

[18] Falconer D.S., Mackay T.F.C. (1996). Introduction to Quantitative Genetics.

[19] Prigge V., Sanchez C., Dhillon B.S., Schipprack W., Araus J.L., Bänziger M., Melchinger A.E. (2011). Doubled haploids in tropical maize: I. Effects of inducers and source germplasm on in vivo haploid induction rates. Crop Sci..

[20] Semagn K., Babu R., Hearne S., Olsen M. (2014). Single nucleotide polymorphism genotyping using Kompetitive Allele Specific PCR (KASP): Overview of the technology and its application in crop improvement. Mol. Breed..

[21] Kebede A.Z., Dhillon B.S., Schipprack W., Araus J.L., Bänziger M., Semagn K., Melchinger A.E. (2011). Effect of source germplasm and season on the in vivo haploid induction rate in tropical maize. Euphytica.

[22] Petroni K., Tonelli C. (2011). Recent advances on the regulation of anthocyanin synthesis in reproductive organs. Plant Sci..

[23] Hallauer A.R., Carena M.J., Miranda Filho J.B. (2010). Quantitative Genetics in Maize Breeding.

[24] Battistelli G.M., Von Pinho R.G., Justus A., Couto E.G.O., Balestre M. (2013). Production and identification of doubled haploids in tropical maize. Genet. Mol. Res..

[25] Vanous K., Jubery T.Z., Ganapathysubramanian B., Lübberstedt T. (2019). Utilization of reduced haploid vigor for phenomic discrimination of haploid and diploid maize seedlings. TPPJ.

[26] Prigge V., Xu X., Li L., Babu R., Chen S., Atlin G.N., Melchinger A.E. (2012). New insights into the genetics of in vivo induction of maternal haploids, the backbone of doubled haploid technology in maize. Genetics.

[27] Xu C., Ren Y., Jian Y., Guo Z., Zhang Y., Xie C., Fu J., Wang H., Wang G., Xu Y. (2017). Development of a maize 55 K SNP array with improved genome coverage for molecular breeding. Mol. Breed..

[28] Gomez K.A., Gomez A.A. (1984). Statistical Procedures for Agricultural Research.

