# Supplementary material for: Testcross Analysis of Pl-1 Marker Expression and Seedling Vigor in Thai Maize Germplasm for Doubled Haploid Breeding Applications
Source: Plants (Basel). 2025 Sep 29;14(19):3011. doi: 10.3390/plants14193011 (PMC12526231; doi:10.3390/plants14193011)
Supplement: Supplementary file 1 [file plants-14-03011-s001.zip › plants-3889436-supplementary.pdf]

Table S1. Individual genotype performance for *Pl-1* expression and seedling vigor traits.

Genotype ID	Market Class	*Pl-1* Score	Root Length (cm)	Fresh Weight (g)
Field Maize				
F01	Field	1.6	6.2 ± 0.3	1.3 ± 0.1
F02	Field	2.1	7.9 ± 0.4	1.1 ± 0.3
F03	Field	1.6	6.2 ± 0.7	1.4 ± 0.2
F04	Field	1.7	6.3 ± 0.5	1.1 ± 0.1
F05	Field	1.5	5.6 ± 0.3	1.2 ± 0.2
F06	Field	1.7	7.4 ± 0.3	1.8 ± 0.1
F07	Field	0.5	2.5 ± 0.4	0.4 ± 0.0
F08	Field	1.4	6.9 ± 0.7	1.3 ± 0.2
F09	Field	1.9	9.0 ± 0.2	1.1 ± 0.2
F10	Field	1.6	7.0 ± 0.5	1.2 ± 0.1
F11	Field	1.6	7.6 ± 0.3	1.3 ± 0.1
F12	Field	1.8	10.9 ± 0.5	2.3 ± 0.2
F13	Field	2.0	8.3 ± 0.4	2.3 ± 0.1
F14	Field	2.1	7.2 ± 0.5	1.5 ± 0.1
F15	Field	2.0	8.4 ± 0.5	1.6 ± 0.2
F16	Field	1.7	8.0 ± 0.5	1.2 ± 0.1
F17	Field	1.5	5.7 ± 0.8	0.5 ± 0.2
F18	Field	1.4	3.9 ± 0.6	0.7 ± 0.1
F19	Field	1.8	5.9 ± 0.4	1.3 ± 0.1
F20	Field	2.0	8.5 ± 0.3	2.3 ± 0.1
F21	Field	1.4	6.1 ± 0.7	1.1 ± 0.2
F22	Field	1.0	3.5 ± 0.2	0.3 ± 0.1
F23	Field	2.0	6.2 ± 0.4	2.0 ± 0.2
F24	Field	1.8	5.3 ± 0.1	1.3 ± 0.3
F25	Field	1.7	7.0 ± 0.6	1.6 ± 0.3
F26	Field	1.9	7.0 ± 0.5	1.6 ± 0.2
F27	Field	1.6	7.6 ± 0.9	1.7 ± 0.1
F28	Field	1.7	6.5 ± 0.6	1.6 ± 0.1
F29	Field	0.8	1.8 ± 0.5	0.3 ± 0.1
F30	Field	1.4	4.8 ± 0.7	0.4 ± 0.2
Waxy Maize				
W01	Waxy	2.1	8.1 ± 0.1	1.7 ± 0.2
W02	Waxy	1.7	6.4 ± 0.4	1.1 ± 0.2
W03	Waxy	2.3	7.3 ± 0.6	1.8 ± 0.1
W04	Waxy	1.8	6.1 ± 0.5	1.3 ± 0.2
W05	Waxy	2.0	7.0 ± 0.4	1.6 ± 0.1
W06	Waxy	1.9	7.2 ± 0.5	1.4 ± 0.1
W07	Waxy	1.4	5.3 ± 0.5	1.3 ± 0.1
W08	Waxy	2.3	8.4 ± 0.6	2.0 ± 0.1
W09	Waxy	2.0	9.0 ± 0.5	1.6 ± 0.2
W10	Waxy	1.8	8.7 ± 0.4	2.3 ± 0.2
W11	Waxy	1.5	5.2 ± 0.4	1.0 ± 0.1
W12	Waxy	3.0	8.8 ± 0.2	2.9 ± 0.2

Genotype ID	Market Class	*Pl-1* Score	Root Length (cm)	Fresh Weight (g)
W13	Waxy	1.9	8.3 ± 0.5	1.4 ± 0.3
W14	Waxy	1.3	2.5 ± 0.5	0.4 ± 0.2
W15	Waxy	2.6	8.0 ± 0.6	1.7 ± 0.1
W16	Waxy	2.4	8.1 ± 0.4	1.4 ± 0.3
W17	Waxy	2.3	9.3 ± 0.4	1.5 ± 0.2
W18	Waxy	2.3	6.7 ± 0.2	1.0 ± 0.1
W19	Waxy	2.9	8.0 ± 0.7	1.4 ± 0.2
W20	Waxy	2.0	10.5 ± 0.4	2.4 ± 0.3
W21	Waxy	2.0	8.1 ± 0.6	2.3 ± 0.2
W22	Waxy	2.2	7.3 ± 0.8	1.7 ± 0.3
W23	Waxy	2.5	6.6 ± 0.2	1.5 ± 0.1
W24	Waxy	1.5	7.0 ± 0.4	1.3 ± 0.3
W25	Waxy	1.9	8.2 ± 0.5	1.6 ± 0.2
W26	Waxy	1.6	3.8 ± 0.5	1.2 ± 0.2
W27	Waxy	2.3	9.0 ± 0.7	1.8 ± 0.1
W28	Waxy	2.0	7.8 ± 0.1	1.7 ± 0.2
W29	Waxy	2.0	9.6 ± 0.1	2.5 ± 0.2
Sweet Maize				
SC01	Sweet	1.9	5.9 ± 0.4	1.6 ± 0.2
SC02	Sweet	1.4	3.5 ± 0.4	0.7 ± 0.1
SC03	Sweet	1.7	5.7 ± 0.4	1.2 ± 0.1
SC04	Sweet	0.8	3.3 ± 0.5	0.6 ± 0.1
SC05	Sweet	1.4	5.1 ± 0.8	1.2 ± 0.1
SC06	Sweet	1.4	8.0 ± 0.7	1.4 ± 0.2
SC07	Sweet	1.4	2.6 ± 0.4	0.5 ± 0.1
SC08	Sweet	1.7	5.8 ± 0.3	1.1 ± 0.1
SC09	Sweet	2.0	10.4 ± 0.3	2.0 ± 0.3
SC10	Sweet	1.4	5.6 ± 0.5	0.9 ± 0.1
SC11	Sweet	1.3	4.1 ± 0.9	0.6 ± 0.1
SC12	Sweet	1.1	3.8 ± 0.5	0.5 ± 0.1
SC13	Sweet	1.7	7.9 ± 0.5	1.0 ± 0.1
SC14	Sweet	1.7	8.4 ± 0.5	1.8 ± 0.1
SC15	Sweet	1.9	6.7 ± 0.5	1.3 ± 0.3
SC16	Sweet	2.0	7.9 ± 0.7	2.2 ± 0.2
SC17	Sweet	1.8	8.4 ± 0.7	1.1 ± 0.2
SC18	Sweet	1.3	7.7 ± 0.3	0.8 ± 0.1
SC19	Sweet	2.2	6.8 ± 0.8	2.3 ± 0.2
SC20	Sweet	1.5	6.6 ± 0.4	2.0 ± 0.1
SC21	Sweet	1.8	7.6 ± 0.5	1.7 ± 0.0
SC22	Sweet	1.2	6.3 ± 0.3	0.6 ± 0.2
SC23	Sweet	1.6	6.4 ± 0.5	1.1 ± 0.1
SC24	Sweet	1.2	5.4 ± 0.9	1.1 ± 0.2
SC25	Sweet	1.7	6.1 ± 0.2	1.6 ± 0.1
SC26	Sweet	2.2	10.4 ± 0.8	2.1 ± 0.2
SC27	Sweet	3.0	11.0 ± 0.4	2.1 ± 0.3
SC28	Sweet	1.9	9.3 ± 0.8	1.6 ± 0.2

Genotype ID	Market Class	*Pl-1* Score	Root Length (cm)	Fresh Weight (g)
SC29	Sweet	1.1	3.8 ± 0.8	1.2 ± 0.3
SC30	Sweet	1.9	6.4 ± 0.4	1.5 ± 0.3
Controls				
BHI306-1	Control	3.9	4.9 ± 0.3	0.5 ± 0.0
BHI306-2	Control	4.0	4.8 ± 0.3	0.5 ± 0.1

Values represent least square means ± standard error from three replications analyzed using a randomized complete block design. Seedling vigor classification based on primary root length at 96 hours post-imbibition: Very Low (<2.0 cm), Low (2.0-4.0 cm), Moderate (4.0-7.0 cm), High (7.0-12.0 cm), Very High (>12.0 cm), following established protocols for haploid identification in maize [11]. *Pl-1* expression evaluated using the standardized 5-point visual scale under controlled lighting conditions (1000 lux fluorescent white light): 5 = intense purple/red anthocyanin coloration throughout primary root, 1 = complete absence of detectable pigmentation [13]. All measurements conducted under standardized growth chamber conditions (24 ± 1°C, 85-90% relative humidity, complete darkness during germination phase). Genotype nomenclature: F = field maize, W = waxy maize, and SC = sweet maize.
